# Functional changes in neural mechanisms underlying post-traumatic stress disorder in World Trade Center responders

**DOI:** 10.1038/s41398-023-02526-y

**Published:** 2023-07-11

**Authors:** Azzurra Invernizzi, Elza Rechtman, Paul Curtin, Demetrios M. Papazaharias, Maryam Jalees, Alison C. Pellecchia, Stephanie Santiago-Michels, Evelyn J. Bromet, Roberto G. Lucchini, Benjamin J. Luft, Sean A. Clouston, Cheuk Y. Tang, Megan K. Horton

**Affiliations:** 1 Department of Environmental Medicine and Public Health, Icahn School of Medicine at Mount Sinai, New York, NY USA; 2 World Trade Center Health and Wellness Program, Renaissance School of Medicine at Stony Brook University, Stony Brook, NY USA; 3 Department of Psychiatry, Renaissance School of Medicine at Stony Brook University, Stony Brook, NY USA; 4 Department of Environmental Health Sciences, Robert Stempel School of Public Health, Florida International University, Miami, FL USA; 5 Department of Medical Surgical Specialties, Radiological Sciences and Public Health, University of Brescia, Brescia, Italy; 6 Department of Medicine, Renaissance School of Medicine at Stony Brook University, Stony Brook, NY USA; 7 Program in Public Health and Department of Family, Population, and Preventive Medicine, Renaissance School of Medicine at Stony Brook University, Stony Brook, NY USA; 8 Department of Radiology and Psychiatry, Icahn School of Medicine at Mount Sinai, New York, NY USA

**Keywords:** Psychiatric disorders, Neuroscience

## Abstract

World Trade Center (WTC) responders exposed to traumatic and environmental stressors during rescue and recovery efforts have a high prevalence of chronic WTC-related post-traumatic stress disorder (WTC-PTSD). We investigated neural mechanisms underlying WTC-PTSD by applying eigenvector centrality (EC) metrics and data-driven methods on resting state functional magnetic resonance (fMRI). We identified how EC differences relate to WTC-exposure and behavioral symptoms. We found that connectivity differentiated significantly between WTC-PTSD and non-PTSD responders in nine brain regions, as these differences allowed an effective discrimination of PTSD and non-PTSD responders based solely on analysis of resting state data. Further, we found that WTC exposure duration (months on site) moderates the association between PTSD and EC values in two of the nine brain regions; the right anterior parahippocampal gyrus and the left amygdala (*p* = 0.010; *p* = 0.005, respectively, adjusted for multiple comparisons). Within WTC-PTSD, a dimensional measure of symptom severity was positively associated with EC values in the right anterior parahippocampal gyrus and brainstem. Functional neuroimaging can provide effective tools to identify neural correlates of diagnostic and dimensional indicators of PTSD.

## Introduction

The men and women involved in the rescue and recovery efforts following the 9/11 World Trade Center (WTC) tragedy were exposed to a complex mixture of smoke, dust and debris generated by the collapse and lasting fires of WTC buildings [1]. Concurrently, WTC responders experienced traumatic psychosocial stressors including fear for personal safety, injury or illness, exposure to human remains, working long hours and performing arduous work in chaotic conditions [1]. Twenty years later, approximately 23% of WTC responders continue to experience chronic posttraumatic stress disorder (PTSD) [2–4], a psychiatric disorder characterized by persistent and intrusive memories of the stressful events at the WTC that can cause behavioral and social impairments.

Recent studies of WTC-responders have used structural MRI to characterize anatomical differences between WTC responders who did (WTC-PTSD) or did not develop PTSD (non-PTSD) [5, 6]. These studies have demonstrated evidence of anatomical changes such as reduced cortical complexity across brain areas (frontal, parietal, and temporal) [6], and have noted heightened glial activation in responders with more severe PTSD symptomatology [7]. The magnitude of structural changes was associated with PTSD symptom severity across four symptom domains re-experiencing, avoidance, hyperarousal, and negative thoughts [6]. To date, no studies have used functional MRI to characterize PTSD in WTC-responders.

Functional magnetic resonance imaging (fMRI) may prove useful in understanding, detecting, and monitoring neurobiological mechanisms underpinning the associations between traumatic psychological and environmental exposures such as 9/11 and PTSD. Spontaneous (task-independent) signal fluctuations observed during resting-state fMRI (rs-fMRI) have been widely used to investigate functional alterations in cortical and subcortical brain areas in psychiatric disorders [8] and to understand underlying mechanisms of PTSD [8, 9]. The existing fMRI and rs-fMRI studies suggest that PTSD follows the ‘fear-conditioning’ paradigm [10–12] characterized by exaggerated amygdala responses and reduced functionality in frontal lobe and hippocampal regions [12–15]. Despite the many studies that investigate PTSD, little is still known about unique populations, like WTC responders and how PTSD impacts them.

Graph theory models leveraging rs-fMRI data provide a comprehensive set of quantitative measures including network centrality that can be used to investigate global (network-wide) and local (network-specific) aspects of neural connectivity. For example, among participants with PTSD, the influence that a specific brain region has on system-wide information flow and integration as measured using neural centrality has previously been found to be reduced in hierarchical brain networks [16–18]. Centrality metric spatially characterizes the connectivity and contribution of each single brain region in dynamic network processes captured by function MRI data [19]. By using this graph metric, it is possible to identify local and specific changes in neural activation among individuals with PTSD, a crucial step to advance intervention guidelines and create treatment protocols such as non-invasive brain stimulation, to modulate brain activity and elicit behavioral changes [20–23]. Prior efforts have yet to examine the role of centrality in relation to PTSD. Furthermore, selective local changes of centrality identified in individuals with PTSD might produce maladaptive behaviors characterized by the set of PTSD specific symptoms including re-experiencing (i.e. involuntary intrusive memories, flashbacks, etc.), avoidance (of distressing thoughts, feelings trauma-related inputs), altered arousal and reactivity (hyperarousal that includes irritability, aggressive and self-destructive behaviors, concentration and sleep problems) and recurrently negative thoughts [8–10].

Here, we leverage network centrality to understand functional changes in neural mechanisms underlying WTC-PTSD, and to identify selective differences in local brain areas that are associated with the WTC-exposure. Specifically, we hypothesized that eigenvector centrality (EC) [19] derived from rs-fMRI data [24, 25] could be combined with advanced data-driven methods to: (a) discriminate and identify differences in connectivity between WTC-PTSD and non-PTSD responders; (b) link these differences to measures of WTC-exposures; and (c) examine how these changes in centrality associate with PTSD symptom scales. We focused on highly connected (or disconnected) nodes (i.e., hubs of differential connectivity) that may help to direct and facilitate information flow and integration globally. Based on local EC differences between WTC-responders with and without PTSD, we define hubs of differential connectivity as key areas to investigate the effect of WTC-exposure on the brain. As an exploratory aim of this study, we examined the association between PTSD symptom scales and the EC values in these hubs. This study will expand our knowledge of the biological mechanisms and underlying changes in plasticity of the human brain in WTC-responders that experienced the traumatic exposures at 9/11.

## Methods and materials

### Participants

Ninety-nine participants were recruited from a single clinic-based monitoring WTC Health program who previously participated in an epidemiology study of cognitive accelerated aging. Complete details of the study can be found elsewhere [26–29]. Briefly, all participants were aged 44–65 years, fluent in English and satisfied eligibility criteria for MRI scanning (i.e., no prior history of traumatic brain injury, body mass index (BMI) ≤ 40). Within 3 months prior to the MRI scan, global cognitive status was objectively assessed using the Montreal Cognitive Assessment (MoCA) [26]. The diagnostic assessment of PTSD related to WTC (WTC-PTSD) was determined from a structured diagnostic interview, described in detail below. Upon enrollment, WTC-PTSD case and non-PTSD control groups were matched on age within 5 years, sex, race/ethnicity and type of responders (i.e., police) [27, 30]. From the 99 participants who completed the MRI scan, 3 subjects were excluded for poor quality MRI data (i.e., excessive movement, reduced field), leaving 96 participants included in this project. Study procedures that follow the Declaration of Helsinki, were approved by the Institutional Review Boards at both Stony Brook University and the Icahn School of Medicine at Mount Sinai. Informed consents were signed by all participants at enrollment after all study procedures were fully explained.

### Neuropsychological assessment

PTSD diagnosis was assessed using the Structured Clinical Interview for the DSM-IV (SCID-D) [31], a semi-structured interview schedule administered by trained clinical interviewers. Symptom subdomains were measured using continuous subscales calculated using reported symptoms in the SCID for the following subscales: re-experiencing, avoidance, hyperarousal, and negative thoughts symptoms (scores ranging from [10-30], [14-42], [10-30], [8-24], respectively). The PTSD module used WTC exposures as the index trauma. In particular, SCID items were modified to assess PTSD symptoms in relation to traumatic WTC exposure events (i.e. the worst episode of symptoms since 11 September 2001) [30]. Severity is rated on a scale from 1 (not at all) to 5 (extremely) [2]. Then, categorical scores were converted to continuous scores by summing the value of the SCID responses. For example, for the re-experiencing scale, there are 10 items. If all items are a “1”, the score for re-experiencing is equal to 10. If all items on re-experiencing scale are “3”, then the score for re-experiencing is 30. Complete conversion table can be found in supplementary material, Table S1. Eligibility criterion for WTC-PTSD status was presence/absence of current PTSD diagnosis at the time of enrollment into the current study. WTC-PTSD status was considered ‘present’ if criteria were reported in the past month at the time of the interview [30]. Major depressive disorder (MDD) was assessed using the SCID-IV and the presence or absence of current (i.e., active in the past month) MDD diagnosis was determined. MDD was not an exclusion criterion.

### WTC exposure duration

An interviewer-administered exposure assessment questionnaire (EAQ) was administered to all WTC-responders upon enrollment in the CDC/NIOSH supported WTC General Responders Cohort and collected at the first monitoring visit of the epidemiologic study only [28]. Responders were asked to describe the time spent (in months) working on the WTC site [30, 32]. WTC exposure (i.e., #months duration at the WTC site, ranged from 0 to 10 months). This exposure variable was not available for 10 participants, therefore analyzes including this variable were done using a sample of 86 WTC responders. There is no significant difference in demographic characteristics or PTSD status between the participants removed (*n* = 10) and the participants included in the analysis (*n* = 86).

### MRI and fMRI data acquisition

Magnetic resonance imaging (MRI) and functional MRI (fMRI) data acquisition was performed on a high-resolution 3-Tesla SIEMENS mMR Biograph scanner using a 20-channel head and neck coil. For each WTC responder, a high-resolution 3D T1-weighted structural scan was acquired using a MPRAGE sequence (TR = 1900ms, TE = 2.49 ms, TI = 900 ms, flip angle = 9, acquisition matrix = 256 × 256 and 224 slices with final voxel size = 0.89 × 0.89 × 0.89 mm). Then, a single 10-minute continuous functional GE-EPI sequence consisted of gradient-recalled acquisition in the steady state (TR = 1500 ms; TE = 27 ms; pulse angle, 80 degree, field of view = 22 cm, acquisition matrix = 74 × 74 and slice thickness of 2.5 mm) was acquired, for a total of 400 volumes. Fifty contiguous oblique-axial sections were used to cover the whole brain where the first four images were discarded to allow the magnetization to reach equilibrium. During resting-state scans, participants were instructed to relax, lay awake, not think about anything, lie still, with their eyes open. Padding was used for a balance between comfort and reduction of head motion.

### fMRI data analyses

Image pre-processing, eigenvector centrality mapping (ECM), and statistical analyzes were performed using SPM12 (Wellcome Department of Imaging Neuroscience, London, UK), fastECM toolbox [19] and customized scripts, implemented in MatLab 2016b (The Mathworks Inc., Natick, Massachusetts) and R (v3.4).

#### Image preprocessing

For each subject, the structural magnetic resonance image was co-registered and normalized against the Montreal Neurological Institute (MNI) template and segmented to obtain white matter (WM), gray matter (GM) and cerebrospinal fluid (CSF) probability maps in the MNI space. FMRI data were spatially realigned, co-registered to the MNI-152 EPI template and subsequently normalized utilizing the segmentation option for EPI images in SPM12. All normalized data were denoised using ICA-AROMA [33]. Additionally, spatial smoothing was applied (8 millimeters) to the fMRI data. No global signal regression was applied.

Based on the Harvard-Oxford [34, 35] atlas currently distributed with the FMRIB software library (FSL [36, 37], https://fsl.fmrib.ox.ac.uk/fsl/fslwiki/FSL), 111 regions of interest (ROI; 48 left and 48 right cortical areas; 7 left and 7 right subcortical regions and 1 brainstem) were defined. In this atlas, the brain areas were defined using T1-weighted images of 21 healthy male and 16 healthy female subjects (ages 18–50). The T1-weighted images were segmented and affine-registered to MNI152 space using FLIRT (FSL), and the transforms then applied to the individual brain areas’ label. Finally, these labels were combined across subjects to form population probability maps for each ROI. Note that the use of the Havard-Oxford parcellation constrains the identification of nodes, increasing the generalizability of our methodology.

For each ROI identified, a time-series was extracted by averaging across voxels per time point. Then, to facilitate statistical inference, data were “pre-whitened” by removing the estimated autocorrelation structure in a two-step GLM procedure [38, 39]. In the first step, the raw data were filtered against the 6 motion parameters (3 translations and 3 rotations). Using the resulting residuals, the autocorrelation structures present in the data were estimated using an Auto-Regressive model of order 1 (AR(1)) and then removed from the raw data. Next, the realignment parameters, white matter (WM) and cerebrospinal fluid (CSF) signals were removed as confounders on the whitened data.

#### Eigenvector centrality mapping (ECM)

Eigenvector centrality mapping (ECM) is a measure to spatially characterize connectivity in functional brain imaging by attributing network properties to voxels [40–43]. The ECM method builds on the concept of eigenvector centrality, which characterizes functional networks active over time and attributes a voxel-wise centrality value to each ROI. Such a value is strictly dependent on the sum of centrality properties of the direct neighbor ROI within a functional network. In our study, fast ECM (fECM) [19] toolbox, an efficient algorithm was used to estimate voxel-wise eigenvector centralities from the time course data extracted based on the Harvard-Oxford ROIs definition per subject. ECM is estimated from the adjacency matrix, which contains the pairwise correlation between the ROIs. To obtain a real-valued EC value, we added +1 to the values in the adjacency matrix. Several EC values can be attributed to an individual node by the ECM method [19], but only the eigenvector with the highest eigenvalue (EV) will be used for further analyzes for each node. The highest EV values were averaged across subjects at group level.

### Statistical analyzes

#### Descriptive statistics

By study design, sample subgroups (WTC-PTSD and non-PTSD) were matched for age at the time of the visit, sex, race/ethnicity and education level [2, 6, 28, 30]. Pairwise Student *t*-tests with Welch’s correction for continuous variables and *χ*_2_ tests for categorical variables were used to examine differences in clinical and demographic characteristics across the groups.

#### Permutation statistics

We quantify possible hubs of differential connectivity by comparing the EC values across groups using a family-wise error corrected (FWE) permutation test. Permuted labels based on group definitions (WTC-PTSD vs. non-PTSD) were repeated 1000 times per subject, *p* ≤ 0.05 was considered statistically significant. Only ROIs with EC values that differ significantly between groups were considered influential functional ROIs, i.e. hubs [44–46]. FWE correction was applied for the number of group level comparisons and for the total number of ROIs analyzed. Only *p* values FWE corrected and adjusted for use of medications (psychotropic and opioids) and current depression (MDD) are reported.

#### General linear model

To test our hypothesis that WTC exposure duration (months on site) moderate the association between PTSD and EC values in hubs of differential connectivity, general linear model (GLM) regressions were computed using current PTSD diagnosis and cumulative WTC exposure duration expressed in months as predictors and EC values for each region as outcomes. These models were adjusted for the use of medications (psychotropic and opioids) and current depression diagnosis. Only EC values of brain areas identified as hubs in permutation analyzes were entered as outcomes in this analysis. GLMs were implemented using R (Version 1.4.1717).

#### Generalized weighted quantile sum regression

To determine associations between EC in the hubs of differential connectivity and PTSD symptoms, we used weighted quantile sum (WQS) regression [47]. WQS is a data driven, mixtures-based ensemble modeling strategy that tests for associations between the combined effect of multiple, correlated variables and an outcome of interest. While developed as a chemical mixtures-based strategy, prior studies demonstrate the utility of WQS for modeling social and behavioral exposures, as well as for the assessment of integrated measures of functional connectivity [48, 49]. Here, we include four dimensional scales of PTSD symptoms as predictors: re-experiencing, avoidance, hyperarousal, and negative thoughts. The WQS analysis is implemented in two steps. First, a weighted index representing the association between each individual dimensional scales of PTSD and EC was estimated across 5000 bootstrap samples. Second, this weighted index was tested in a linear regression model predicting the association between the “mixture” of the PTSD scales and EC. Prior to model estimation, all exposures were quartiles. The mixture of PTSD symptom scale is defined such that WQS = $\mathop {\sum}\nolimits_{i = 1}^c {\quad w_iq_{i,j}}$ is the sum of the cross products of the empirically estimated weight (*w*_*i*_) for each predictor variable (*i*) and the ranked concentration of that predictor per subject (*q*_*i,j*_). A significance test for the WQS index provides an estimate of the association with the overall PTSD symptom scales, while the weights associated with each predictor provide an indicator of each individual variable’s contribution to the overall effect. All weights are constrained to sum to one, enabling sorting by relative importance. Factors that impact the outcome have larger weights; factors with little or no impact on the outcome have near-zero weights. Only WTC-PTSD responders were included in this analysis. These models were adjusted for use of medications (psychotropic and opioids) and current depression (MDD).

## Results

### Demographic and clinical characteristics

Table 1 reports the clinical and demographic characteristics for the 96 WTC responders included in this study stratified by responders with PTSD (WTC-PTSD) and without PTSD (non-PTSD). Responders were in their mid-fifties at the time of the imaging data acquisition (55.8 ± 5.2 years) and the majority were male (79%). By design, groups were matched on age at the time of the neuroimaging scanning, sex, race/ethnicity, and educational attainment. Current major depression diagnosis (MDD), daily use of psychotropic medications, and PTSD symptoms scales (DSM-IV SCID trauma screen) significantly differ between groups. No significant difference in WTC-exposure duration was found between WTC responders with/without PTSD (average month on site for WTC-PTSD = 3.87 and non-PTSD = 4.07, *p* = 0.781). Additional characteristics not included in analysis (i.e., ethnicity, occupation, and education level) are reported in supplementary material (Table S2). Table 1 and S2 indicate the group matching WTC-PTSD and non-PTSD was successful and therefore, further analyses were not adjusted for variables like age, sex, race/ethnicity, and educational attainment.

**Table 1:** Sociodemographic and clinical characteristics of WTC responders who were selected into the current study (*N* = 96).

Characteristics	All WTC responders (*n* = 96)	WTC-PTSD (*n* = 45)	non-PTSD (*n* = 51)	*p*
Age (n)				0.383
mean ± sd	55.81 ± 5.26	55.31 ± 5.18	56.25 ± 5.34	
Sex (n, %)				0.827
Male	76 (79%)	37 (49%)	39 (51%)	
Female	20 (21%)	8 (40%)	12 (60%)	
MoCA (n)				0.736
mean, [range]	23.23 [12,30]	23, [12,30]	23.4 [15,30]	
Comorbidities (n, %)				
Major depressive disorder				<0.001*
No	78 (81%)	27 (60%)	51 (100%)	
Yes	18 (19%)	18 (40%)	0 (0%)	
Cognitive impairment				0.424
No	49 (51%)	22 (43%)	27 (53%)	
Yes	47 (49%)	23 (45%)	24 (47%)	
Medications (n, %)				
Pscychotropic				<0.001*
No	74 (77%)	27 (60%)	47 (92%)	
Yes	22 (23%)	18 (40%)	4 (8%)	
Opioid				0.643
No	92 (96%)	43 (95%)	49 (96%)	
Yes	4 (4%)	2 (5%)	2 (94%)	
WTC Exposure (mean ± sd)				0.853
Total months on site	2.63 ± 5.1	2.73 ± 4.96	2.54 ± 5.2	
DSM-IV SCID Trauma Screen (mean ± sd)				
Re-experiencing	17.46 ± 6.74	23.4 ± 4.35	12.22 ± 3.14	<0.001*
Avoidance	24.01 ± 9.53	32.73 ± 6.12	16.31 ± 3.25	<0.001*
Hyperarousal	17.71 ± 6.67	23.97 ± 3.27	12.17 ± 2.94	<0.001*
Negative thoughts	12.19 ± 4.61	15.75 ± 4.28	9.05 ± 1.66	<0.001*

Mean, standard deviation (sd), range (minimum and maximum values), and percentage (%) are reported. *P* values quantify differences between WTC-PTSD and non-PTSD were derived using Student *t* tests for continuous variables and *χ*_2_ tests for categorical variables. *p < 0.005.

### Centrality differences between WTC-PTSD and non-PTSD

**Fig. 1:**
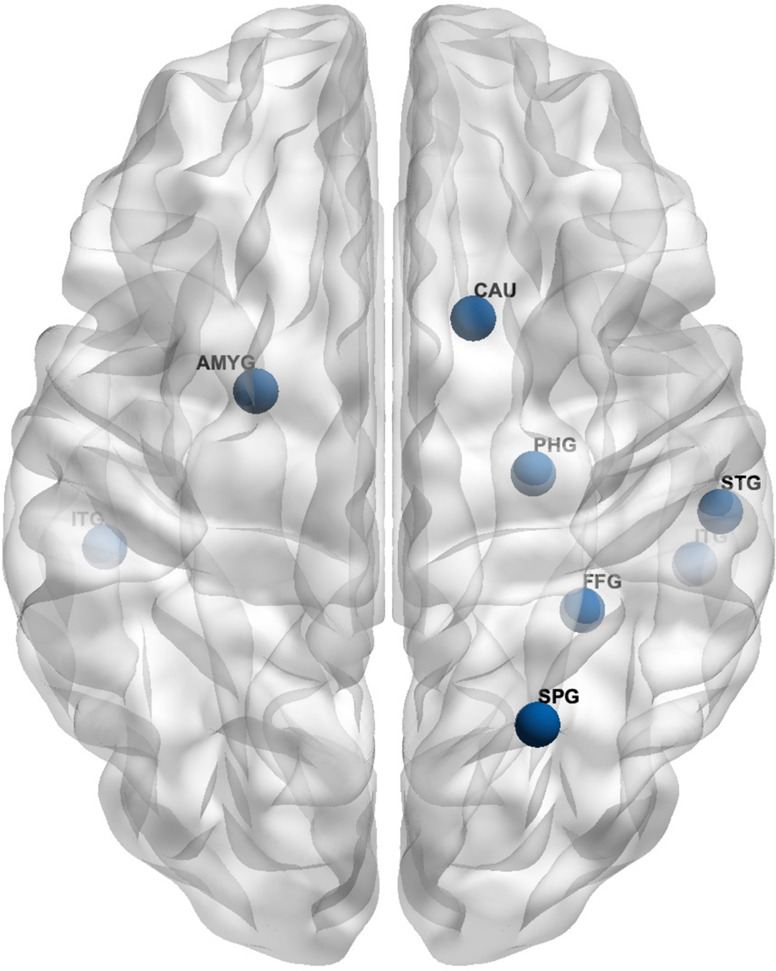
Brain regions differ in eigenvector centrality between WTC-PTSD and non-PTSD. Figure 1 identified ROI for which eigenvector centrality (EC) differed significantly between WTC-responders with and without PTSD (i.e., hubs of differential connectivity).

EC values in permutation tests (non-PTSD > PTSD) revealed nine hubs where EC values differed significantly between WTC-PTSD and non-PTSD groups including: right and left anterior inferior temporal gyrus, right superior parietal lobule, right anterior parahippocampal gyrus, right anterior and posterior temporal fusiform cortex, right caudate nucleus, left amygdala and the brainstem (Table 2, Fig. 1). Notably, of the nine hubs of differential connectivity, seven were localized in the right hemisphere.

**Table 2:** Statistical differences in eigenvector centrality between WTC-PTSD and non-PTSD.

PTSD- versus PTSD+			
Brain region (ROIs)	Hemisphere	Abbreviation	*p*
Inferior Temporal Gyrus (anterior)	R	ITG	0.042
Superior Parietal Lobule	R	SPG	0.018
Parahippocampal Gyrus (anterior)	R	PHG	0.048
Temporal Fusiform Cortex (anterior)	R	FFG	0.012
Temporal Fusiform Cortex (posterior)	R	STG	0.017
Caudate nucleus	R	CAU	0.015
BrainStem	–	–	0.016
Inferior Temporal Gyrus (anterior)	L	ITG	0.015
Amygdala	L	AMYG	0.014

Table 2 identified ROI for which eigenvector centrality (EC) differed significantly between WTC-responders with and without PTSD (i.e., hubs of differential connectivity). The table reports the full name of the brain region of interest (ROIs), ROI’s hemisphere, ROI’s abbreviation, and *p* values of the identified ROIs for which eigenvector centrality (EC) differed significantly between WTC-responders with and without PTSD (i.e., hubs of differential connectivity). Only *p* values FWE corrected, adjusted for the use of medications (psychotropic and opioids), current depression diagnosis (MDD) and <0.005 are reported.

### Centrality and WTC Exposure

**Fig. 2:**
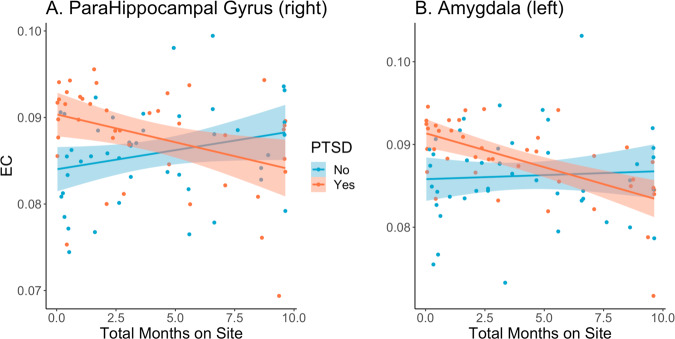
WTC exposure duration and EC values. These graphs plot the relationship (interaction) between WTC exposure duration in months (x-axis) and eigenvector (EC) values (y-axis) stratified by WTC-PTSD (orange dots) and non-PTSD (blue dots) for the right anterior parahippocampal gyrus (**A**) and the left amygdala (**B**). WTC exposure duration (months on site) moderates the association between PTSD status and EC values in both hubs (p_Parahippocampal_ Gyrus= 0.010 and p_Amygdala_ = 0.005).

Results from generalized linear modeling suggest that WTC exposure duration (months on site) moderates the association between PTSD and EC values in two of the nine hubs of differential connectivity; the right anterior parahippocampal gyrus and the left amygdala (p-value for interaction = 0.010 and 0.005, respectively, adjusted for multiple comparisons; Table 3, Fig. 2). In WTC-PTSD responders, prolonged WTC exposure is associated with decreased EC values in these two brain areas, (*p* = 0.05 and 0.002, 95% CI [–0.0005, 0.001; –0.0003, –0.0006], respectively). For completeness, remaining hubs, and models uncorrected for family-wise error rate were reported in supplementary materials (Table S2, Fig. S1, Table S3).

**Table 3:** Association between EC values, PTSD status and WTC exposure duration.

Parahippocampal Gyrus (anterior,right)				Amygdala (left)			
*Predictors*	*Estimates*	*CI*	*p*	*Predictors*	*Estimates*	*CI*	*p*
Intercept	0.084	0.082–0.087	<0.001	Intercept	0.086	0.084–0.088	<0.001
Months on site	0.000	0–0.001	0.099	Months on site	0.000	0–0.001	0.492
Psychotropic	–0.002	0.005–0.002	0.307	Psychotropic	–0.002	0.005–0.000	0.063
Opioid	–0.002	0.008–0.005	0.576	Opioid	–0.008	0.014–0.003	0.001
MDD	–0.002	0.005–0.002	0.367	MDD	0.002	0.001–0.005	0.109
PTSD	0.007	0.003–0.011	<0.001	PTSD	0.005	0.002–0.008	0.001
Months on site*PTSD	–0.001	0.002–0.000	0.01	Months on site*PTSD	–0.001	0.001–0.000	0.005
Observations	86				86		
R2	0.162				0.332		

Generalized linear regression models (GLM) examining WTC exposure duration (i.e., months on site) moderates the association between PTSD (WTC-PTSD versus non-PTSD) and EC values controlling for major depressive disorder (MDD) and medication use (psychotropic and opioid) on eigenvector centrality (EC) value of a single brain area (defined using the Harvard-Oxford atlas). *P* values are adjusted for multiple comparisons.

### Centrality and PTSD symptoms

**Fig. 3:**
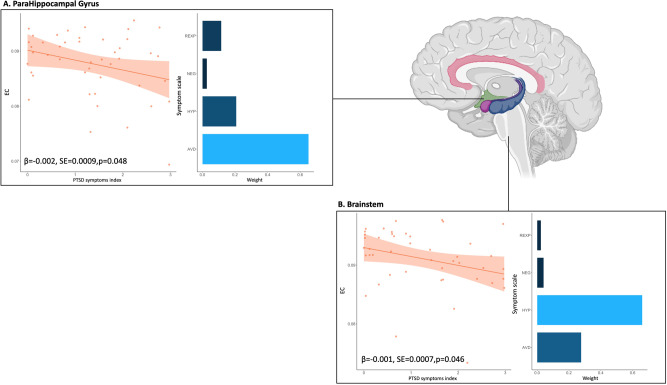
PTSD-symptom scales and centrality. WQS regression analysis of the association between weighted index of PTSD symptoms and EC values for the right anterior parahippocampal gyrus and the brainstem (panel **A**, **B**, respectively). EC values of the right and left anterior inferior temporal gyrus have been averaged into one unique ROI (anterior inferior temporal gyrus) for this analysis. In the graph, orange dots represent WTC-PTSD responders, the orange line represents the association between the PTSD symptoms index and EC values, shaded orange is the confidence interval. The histogram displays the contribution of each PTSD symptom to this association and each bar represents a different symptom scale. Symptoms with the highest weights are plotted in light blue. WQS values are reported in Table 4.

Within the WTC-PTSD group, we identified significant associations between EC values and the weighted PTSD symptom index in the right anterior parahippocampal gyrus (β = –0.002, SE = 0.0009, *p* = 0.048; Table 4) and the brainstem (β = –0.001, SE = 0.0007, *p* = 0.046; Table 4). Avoidance symptoms contributed 64.3% to the overall association between EC values and PTSD symptoms index in the right anterior parahippocampal gyrus (Fig. 3A). Hyperarousal contributed 65.4% to this association in the brainstem (Fig. 3B). None of these associations survived correction for multiple comparisons.

**Table 4:** PTSD-symptom scales and centrality.

Brain region (ROI)	β	SE	*p*	p-corrected
Inferior Temporal Gyrus (anterior)	–0.001	0.0008	0.117	0.234
Superior Parietal Lobule (right)	0.001	0.0008	0.292	0.334
Parahippocampal Gyrus (anterior,right)	–0.002	0.0009	0.048	0.163
Temporal Fusiform Cortex (anterior,right)	–0.001	0.0009	0.233	0.311
Temporal Fusiform Cortex (posterior, right)	–0.003	0.0006	0.061	0.163
Caudate nucleus (right)	–0.001	0.0011	0.424	0.424
BrainStem	–0.001	0.0007	0.046	0.162
Amygdala (left)	–0.001	0.0007	0.232	0.311

WQS regression analysis of the association between weighted index of PTSD symptoms and EC values for the right anterior parahippocampal gyrus and the brainstem (panel **A** and **B**, respectively). EC values of the right and left anterior inferior temporal gyrus have been averaged into one unique ROI (anterior inferior temporal gyrus) for this analysis.

## Discussion

The mechanisms through which PTSD impacts neural functioning are not well established. This is the first study to use graph-based network metrics of rs-fMRI and data-driven methods to investigate local connectivity differences between PTSD and non-PTSD in WTC responders, filling the gap in this literature. We identified clear differences in functional neuro-profiles of WTC-responders with and without PTSD providing a robust basis for discriminating trauma survivors that did and did not develop PTSD. Discrimination between groups is primarily attributable to connectivity differences in nine brain regions. WTC exposure duration (months on site) moderates the association between PTSD and EC values in two of the nine hubs of differential connectivity; the right anterior parahippocampal gyrus and the left amygdala, both previously linked to fear conditioning [50, 51] and to PTSD [52–54]. Finally, within the WTC-PTSD group, we observed associations between functional network properties and symptoms reports, in this case the dimensional PTSD symptoms, in the right anterior parahippocampal gyrus and in the brainstem, suggesting associations between functional brain changes and symptoms reports. Our results confirm previous results presented in literature and further contribute to our understanding of the neurobiological underpinnings of PTSD in WTC responders. These results may guide treatment efforts and inform future disaster-response activities.

Seven out of the nine hubs that differ between WTC-PTSD and non-PTSD in our study were located in the right hemisphere (Fig. 1, Table 2), suggesting lateralization of the association between WTC-PTSD and centrality. These results align with our previous study in this cohort demonstrating anatomical changes with strong lateralization in the right hemisphere across several brain areas [6]. Notably, the left hemisphere that is mostly involved with verbal communication and problem-solving abilities, seems to be less associated with PTSD in our study and in others [6, 55]. Taken together, these results of lateralized changes for both functional and structural data in WTC-responders [6] align with previous studies performed in traumatized subjects [55–57], where the right hemisphere appears to be more generally affected by PTSD [6, 55] when compared to the left hemisphere.

Among the more than 35,000 responders enrolled in the ongoing WTC-HP, 23% of them continue to experience chronic WTC-related PTSD [2–4]. In this study of responders selected on PTSD case status (WTC-PTSD vs WTC non-PTSD), the duration of WTC exposure (i.e., number of months spent at the WTC site in rescue and recovery efforts) did not differ between responders with PTSD and those without PTSD (Table 1). While all responders experience some degree of traumatic exposure, not all responders develop PTSD. Among responders with PTSD, decreased connectivity in the right anterior parahippocampal gyrus and the left amygdala, are associated with prolonged WTC exposure during search and rescue efforts at and for months after 9/11 (Fig. 2, Table 3). During these months on the pile, WTC responders experienced traumatic events and inhaled dust and smoke containing many pollutants (i.e., particulate matter, lead, polycyclic aromatic hydrocarbons (PAHs), polychlorinated biphenyls (PCBs), and dioxins). This unique combined exposure may play a role in the anatomical and functional changes observed in our population [58–61]. These changes and their significant association with longer WTC exposure involve only specific cortical and subcortical brain regions such as the whole hippocampus and its subfields [5], parahippocampal gyrus, amygdala, and frontal and parietal brain regions [6] that seem more vulnerable to experience at the WTC site. The fear-conditioning mechanism and novel neurocircuitry models [10–12] suggest the triggering event, in this case WTC responders experienced during rescue and recovery efforts, targets brain areas known to be involved in PTSD, i.e., the parahippocampus and amygdala. In particular, the amygdala plays a key role in in PTSD [52–54] and is involved in personality, emotional, and behavioral regulation [62, 63], fear and fear conditioning [50, 51], and memory of stressful events [64]. Our results are consistent with previous studies in the WTC cohort that found associations between longer WTC exposure and structural changes defined as reduced cortical volumetric and decreased cortical complexity [5, 6].

PTSD is characterized by recurrent, intercorrelated symptoms such as re-experiencing, avoidance, negative affect, and hyperarousal. Neurobiological models of PTSD show that each of these symptoms are associated with changes in specific brain areas [9]. Disentangling the unique contribution of each PTSD symptom within the centrality neuroprofiles in the WTC-PTSD group contributes to our understanding of neurobiological mechanisms underpinning WTC related PTSD. In order to do so, we used an empirically-estimated index of symptom severity (WQS) that was derived from the observed characteristics of the WTC-PTSD cohort. Overall, our findings are generally consistent with the broad patterns of PTSD symptomology in the literature [8, 65–71]. In our cohort, avoidance symptoms contributed most to the association between overall PTSD symptoms cores and EC shifts in the parahippocampal gyrus. The parahippocampal gyrus surrounds the hippocampus and is part of the temporal lobe network. Parahippocampal gyrus function is crucial for encoding and retrieving episodic, spatial, and contextual memories [65–69]. Consistent with these functions, previous studies linked this cortical brain area with avoidance behavior, disrupted encoding of episodic and autobiographical memories and functional changes in PTSD subjects [66, 72]. Hyperarousal symptom contributed the most to the association between PTSD symptoms scores and centrality values (Fig. 3). The brainstem is critical to convey continued inputs brain-body and to regulate a number of conscious and unconscious processes (i.e., to generate and maintain the general arousal state and to provide the trigger for innate, reflexive defensive responses [73, 74]). Previous studies showed that prolonged and repeated traumatic experiences lead to changes in this brain area and emerging evidence suggests its critical role in the neurobiological model of PTSD [8, 70, 71]. Taken together, our findings show functional changes and association with WTC-exposure in the brainstem, amygdala, and the parahippocampal gyrus areas in WTC-PTSD responders. Similar to findings from a previous study in these WTC responders, we did not find significant changes in the hippocampus area [5]. Our findings are further consistent with several studies demonstrating the relevance of these cortical and subcortical areas in the innate threat processing-related network, well-connected brain areas responsible for triggering the alert and defense mechanisms by a fast communication between deep- to higher-layer of brain regions, in PTSD and in its subtype [75–77]. Unbalanced and disrupted patterns of communication between the amygdala and parahippocampal gyrus have been previously reported in PTSD [8, 68, 78, 79]. In agreement with these studies [8, 78], we report an exaggerated role of the amygdala in WTC-PTSD. However, EC values can only inform us about the influence of the amygdala and how it is connected to other highly connected ROIs, but it does not define a system-wide network, limiting what meaning can be extrapolated to functional processes like threat processing. Notably, while a previous study within these WTC responders did not report significant structural changes in the hippocampus complex [5], our results highlight the importance of examining functional changes in cortical and subcortical areas in PTSD. Finally, our understanding of the neural mechanisms underlying WTC-PTSD is crucial for the progression of novel treatments and interventions that are still lacking for this disorder [9, 22, 23].

Our work shows how rs-fMRI data together with a reliable functional-connectivity based method advances the identification of brain regions that can potentially be used as targets in customizing intervention treatments. Treatments based on transcranial magnetic stimulation and deep brain stimulation might consider the use of rs-fMRI data together with targeting data-driven methods (i.e. graph theory) to customize intervention by modulating relevant neural networks [9, 23, 25]. It is important to point out that this study looks only at one particular network property (EC) and does not fully characterize the role of these hubs within the broader neurocircuitry associated with PTSD-related symptoms and behaviors. Additional follow up studies are needed to understand the characteristics of these particular nodes within the broader relevant functional networks and to further clarify the specific relevance to the development of PTSD related symptoms and behaviors in WTC responders and the population more broadly.

Limitations of our study design include the small sample size and lack of external control group (non-WTC). Our small sample size prohibited splitting the sample into training and validation subset. A larger sample size might improve statistical power and allow us to identify additional hubs of differential connectivity associated with WTC-PTSD. In addition, while a strong effort was made to increase the recruitment of underrepresented populations including women and people of color to the point of doubling the numbers of both groups in this sample compared to the responder population enrolled in our program [27, 32, 80], our sample could nevertheless benefit from improved diversity in order to facilitate subgroup analyzes that are out of reach of this study. Due to the unique nature of this cohort, our analysis cannot disentangle if the functional differences observed between WTC-PTSD and non-PTSD are due to predisposition, the use of psychotropic medications (or other intensive therapy received only by the PTSD group) or other factors, additional follow up studies are needed. In this study, rs-fMRI data was used to investigate the brain signal of WTC-responders. Besides the low temporal and spatial resolution (~8 mm) [81] that prevents proper neuroanatomical dissection, rs-fMRI has been widely and successfully used in the past decades to investigate the intrinsic functional connectivity of the brain [82, 83]. However, given the low burden that it puts on the participants during data acquisition, rs-fMRI is a powerful tool to investigate the functional cortical processes in health and clinical populations. The exposure questionnaire, from which we gather self-reported experience during WTC rescue and recovery efforts, was often first administered years after 9/11 experiences were completed and may therefore be subject to recall bias. Finally, we lack accurate assessments of life trauma and/or PTSD status and MRI scans in WTC responders prior to 9/11, and we lack a comparison group of responders with subsyndromal, mild, heterogeneous, or remitted PTSD. While these limitations do reduce the generalizability of these findings in the general population, individuals exposed to traumatic circumstances are always different in critical ways from the general population. Nevertheless, these studies have consistently identified symptoms in PTSD across populations that match those reported in this population, supporting the view that results from this study are generalizable to other trauma-exposed populations.

To conclude, this is the first ever study using rs-fMRI data to provide novel insights into the underlying neural mechanisms and changes in plasticity of the human brain in WTC-responders that experienced the traumatic exposures at 9/11. Our results suggest that responders who developed WTC-related PTSD present significant brain functional changes in specific brain areas previously shown to be associated with PTSD. These changes are associated with WTC-exposure, as well as with PTSD symptomatology. Future studies to elucidate the different contributing factors to the etiology of PTSD in WTC responders are still needed to advance our understanding in this debilitating disease and to help intervention and treatment.

## Supplementary information

Supplementary material

## Data Availability

De-identified data and code used in this manuscript to generate the results here presented will be made available upon reasonable request to the corresponding author; raw image files can be accessed upon completion of a data use agreement.
