# Supplementary material for: Functional changes in neural mechanisms underlying post-traumatic stress disorder in World Trade Center responders
Source: Transl Psychiatry. 2023 Jul 11;13:239. doi: 10.1038/s41398-023-02526-y (PMC10333341; doi:10.1038/s41398-023-02526-y)
Supplement: Supplementary file 1 — Supplementary material [file 41398_2023_2526_MOESM1_ESM.pdf]

Supplementary material

Table S1 -SCID conversion table from categorical to continuous scores.

SCID Re-Experience scale	SCID Avoidance Scale	SCID Hyperarousal Scale	SCID Negative Thoughts Scale
A score of 10 indicates Re-Experiencing Symptoms are absent	A score of 14 indicates Avoidance Symptoms are absent	A score of 10 indicates Hyperarousal Symptoms are absent	A score of 8 indicates Negative thoughts are absent
Scores between 11-15 indicates Mild rates of Re-Experiencing symptoms	Scores between 15-21 indicates Mild rates of Avoidance symptoms	Scores between 11-15 indacte Mild rates of Hyperarousal symptoms	Scores between 9-12 indicates Mild rates of Negative thoughts
Scores between 16-20 indicates Moderate rates of Re-Experiencing symptoms	Scores between 22-28 indicates Moderate rates of Avoidance symptoms	Scores between 16-20 indicates Moderate rates of Hyperarousal symptoms	Scores between 13-16 indicates Moderate rates of Negative thoughts
Scores between 21-25 indicates Severe rates of Re-Experiencing symptoms	Scores between 29-35 indicates Severe rates of Avoidance symptoms	Scores between 21-25 indicates Severe rates of Hyperarousal symptoms	Scores between 17-20 indicates Severe rates of Negative thoughts
Scores between 26-30 indicates Extreme rates of Re-Experiencing symptoms	Scores between 36-42 indicates Extreme rates of Avoidance symptoms	Scores between 26-30 indicates Extreme rates of Hyperarousal symptoms	Scores between 21-24 indicates Extreme rates of Negative thoughts

Table S2 -Sociodemographic characteristics of WTC responders who were selected into the current study (N=96). Mean, standard deviation (sd), range (minimum and maximum values), and percentage (%) are reported. P-values quantify differences between WTC-PTSD groups and the noted characteristics; they were derived using Student *t*-tests for continuous variables and χ_2 tests for categorical variables. *denotes significance of *p*<0.05.

Characteristics	All WTC responders (n=96)	PTSD- (n=51)	PTSD+ (n=45)	p
**Ethnicity (n,%)**				0.804
White	73 (76.04%)	38 (74.51%)	35 (77.7%)	
Black	10 (10.42%)	6 (11.76%)	4 (8.88%)	
Other	13 (13.54%)	7 (13.72%)	6 (13.33%)	
**Occupation (n,%)**				0.725
NYPD	56 (58.33%)	35 (68.63%)	21 (46.66%)	
Other	38 (39.58%)	15 (29.41%)	23 (51.1%)	
**Education (n,%)**				0.34
High school or less	22 (22.92%)	9 (17.65%)	13 (28.88%)	
College	31 (32.29%)	14 (27.45%)	17 (37.78%)	
University degree	43 (44.79%)	28 (54.90%)	15 (33.33%)	

Table S2 - Association between EC values, PTSD status and WTC exposure duration. Generalized regression models (GLM) examining WTC exposure duration (i.e., months on site) moderates the association between PTSD (WTC-PTSD vs non-PTSD) and EC values controlling for major depressive disorder (MDD) and medication use (psychotropic and opioid) on eigenvector (EC) value of a single brain area (defined using the Harvard-Oxford atlas).

	**Inferior Temporal Gyrus (ant)**			**Superior Parietal Lobule (right)**			**Temporal Fusiform Cortex (anterior,right)**		
Predictors	*Estimates*	*CI*	*p*	*Estimates*	*CI*	*p*	*Estimates*	*CI*	*p*
Intercept	0.086	0.083 – 0.088	**<0.001**	0.095	0.092 – 0.098	**<0.001**	0.082	0.079 – 0.085	**<0.001**
Months on site	-0.000	-0.001 – 0.000	0.841	-0.000	-0.001 – 0.000	0.296	0.001	0.000 – 0.001	**0.042**
psychotropic	-0.001	-0.004 – 0.001	0.315	0.001	-0.002 – 0.005	0.422	-0.001	-0.005 – 0.002	0.407
opioid	-0.004	-0.010 – 0.002	0.155	-0.000	-0.007 – 0.007	0.999	-0.008	-0.015 – -0.001	**0.017**
MDD	0.000	-0.003 – 0.003	0.972	-0.000	-0.004 – 0.003	0.811	0.000	-0.003 – 0.004	0.910
PTSD	0.002	-0.001 – 0.006	0.196	-0.000	-0.004 – 0.004	0.901	0.005	0.001 – 0.009	**0.008**
Months on site*PTSD	-0.000	-0.001 – 0.001	0.862	0.001	-0.000 – 0.001	0.105	-0.001	-0.001 – 0.000	0.061
Observations	86			86			86		
R^2	0.077			0.102			0.156		

	**Temporal Fusiform Cortex (posterior,right)**			**Caudate (right)**			**BrainStem**		
Predictors	*Estimates*	*CI*	*p*	*Estimates*	*CI*	*p*	*Estimates*	*CI*	*p*
Intercept	0.087	0.085 – 0.090	**<0.001**	0.088	0.085 – 0.091	**<0.001**	0.089	0.086 – 0.092	**<0.001**
Months on site	0.000	-0.000 – 0.001	0.591	0.000	-0.000 – 0.001	0.161	-0.000	-0.001 – 0.000	0.821
psychotropic	-0.002	-0.005 – 0.001	0.149	-0.002	-0.005 – 0.002	0.354	0.000	-0.003 – 0.003	0.901
opioid	-0.002	-0.007 – 0.003	0.417	-0.006	-0.013 – 0.001	0.099	-0.001	-0.007 – 0.006	0.812
MDD	-0.002	-0.005 – 0.001	0.173	0.002	-0.002 – 0.006	0.267	-0.001	-0.005 – 0.002	0.401
PTSD	0.004	0.001 – 0.007	**0.015**	0.003	-0.001 – 0.007	0.163	0.003	-0.001 – 0.007	0.105
Months on site*PTSD	-0.000	-0.001 – 0.000	0.639	-0.000	-0.001 – 0.001	0.533	-0.000	-0.001 – 0.001	0.832
Observations	86			86			86		
R^2	0.113			0.115			0.061		

Table S3 - Association between #C values, PTSD status and WTC exposure duration. Series of regression models performed using predictor factors current PTSD diagnosis and, as outcome entered the eigenvector centrality value of a single brain area (defined using the Harvard-Oxford atlas).

	**Inferior Temporal Gyrus (ant)**			**Superior Parietal Lobule (right)**			**ParaHippocampal Gyrus (right)**			**Temporal Fusiform Cortex (anterior,right)**		
Predictors	*Estimates*	*CI*	*p*	*Estimates*	*CI*	*p*	*Estimates*	*CI*	*p*	*Estimates*	*CI*	*p*
Intercept	0.086	0.083 – 0.088	**<0.001**	0.095	0.093 – 0.098	**<0.001**	0.084	0.081 – 0.087	**<0.001**	0.082	0.079 – 0.085	**<0.001**
Months on site	-0.000	-0.001 – 0.000	0.778	-0.000	-0.001 – 0.000	0.240	0.000	-0.000 – 0.001	0.084	0.000	-0.000 – 0.001	0.072
PTSD	0.002	-0.001 – 0.005	0.203	-0.000	-0.004 – 0.003	0.873	0.006	0.003 – 0.010	**0.001**	0.005	0.001 – 0.009	**0.008**
Months on site*PTSD	-0.000	-0.001 – 0.000	0.695	0.001	0.000 – 0.001	**0.049**	-0.001	-0.002 – -0.000	**0.002**	-0.001	-0.001 – -0.000	**0.046**
Observations	86			86			86			86		
R^2	0.038			0.094			0.135			0.082		

	**Temporal Fusiform Cortex (posterior,right)**			**Caudate (right)**			**BrainStem**			**Amygdala (left)**		
Predictors	*Estimates*	*CI*	*p*	*Estimates*	*CI*	*p*	*Estimates*	*CI*	*p*	*Estimates*	*CI*	*p*
Intercept	0.087	0.085 – 0.089	**<0.001**	0.088	0.085 – 0.091	**<0.001**	0.089	0.087 – 0.092	**<0.001**	0.086	0.084 – 0.088	**<0.001**
Months on site	0.000	-0.000 – 0.001	0.553	0.000	-0.000 – 0.001	0.196	-0.000	-0.001 – 0.000	0.778	0.000	-0.000 – 0.001	0.662
PTSD	0.003	-0.000 – 0.006	0.051	0.004	-0.000 – 0.008	0.084	0.003	-0.001 – 0.006	0.148	0.006	0.002 – 0.009	**0.001**
Months on site*PTSD	-0.000	-0.001 – 0.000	0.330	-0.000	-0.001 – 0.000	0.461	-0.000	-0.001 – 0.001	0.818	-0.001	-0.002 – -0.000	**0.003**
Observations	86			86			86			86		
R^2	0.053			0.057			0.052			0.182		

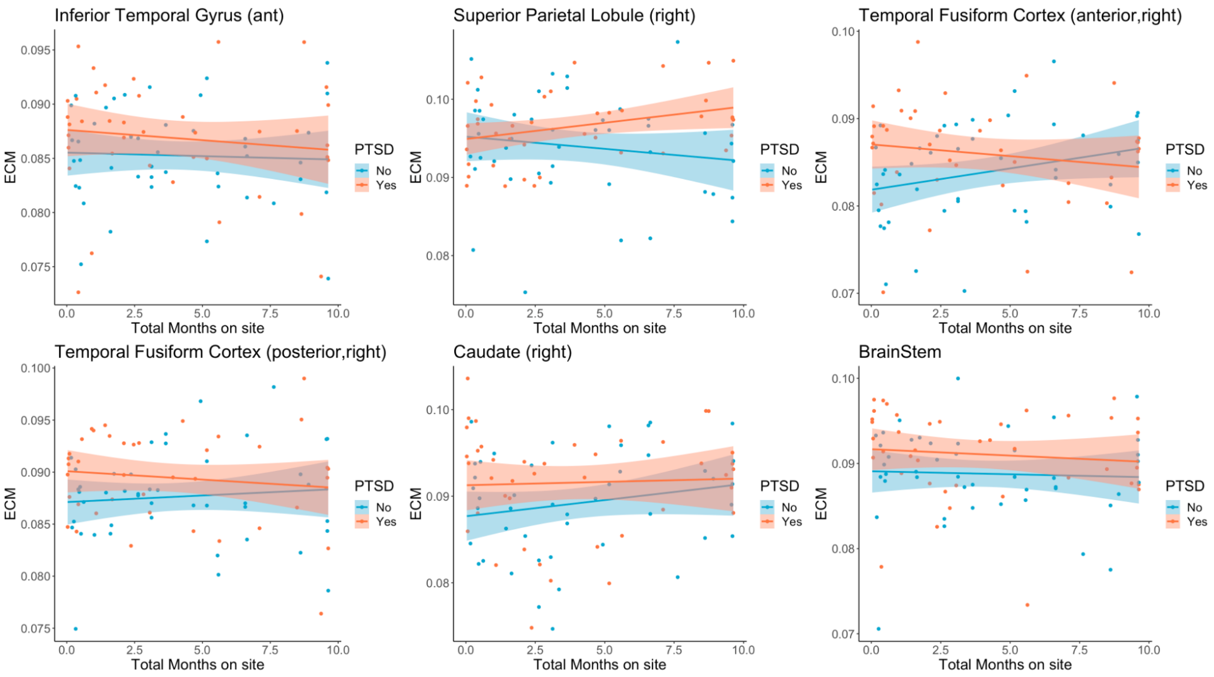

Figure S1 - Effects of WTC exposure duration on centrality values for the identified hubs. These panels show the relation between WTC exposure duration expressed in months and eigenvector centrality values for the nine identified hubs. Orange and blue dots represent WTC-responders with and without PTSD respectively.
